# Causal Associations of Iron Status With the Renal Function and Diabetic Nephropathy in Patients With Diabetes Mellitus: A Two-Sample Mendelian Randomization Study

**DOI:** 10.1155/jdr/6658794

**Published:** 2025-07-30

**Authors:** Ying Zhang, Wujian Peng, Jianrong Huang, Wenchang Zhang, Peishan Jiang, Yuqin He, Meiyun Wang

**Affiliations:** ^1^Department of Endocrinology, Shenzhen Third People's Hospital, The Second Affiliated Hospital, School of Medicine, Southern University of Science and Technology, Shenzhen, Guangdong Province, China; ^2^Department of Nephrology, Shenzhen Third People's Hospital, The Second Affiliated Hospital, School of Medicine, Southern University of Science and Technology, Shenzhen, Guangdong Province, China

**Keywords:** causal effect, diabetic nephropathy, ferritin, iron status, renal function

## Abstract

This study was to explore the causal effect of iron status on renal function and the risk of diabetic nephropathy in diabetic patients. The data on exposures including ferritin, serum iron, transferrin saturation (TSAT), and total iron-binding capacity (TIBC) were obtained from a genome-wide association study (GWAS). The outcomes were diabetic nephropathy, Type 1 diabetes mellitus (T1DM) with renal complications, Type 2 diabetes mellitus (T2DM) with renal complications, estimated glomerular filtration rate (creatinine) (eGFRcrea) in diabetes mellitus, and urinary albumin-to-creatinine ratio (UACR) in diabetes mellitus. The causal associations of exposures and outcomes were analyzed using MR analysis with the inverse variance-weighted (IVW) method as the primary analytical method. Leave-one-out analysis was utilized to find any individual SNP associated with exposures influencing outcomes. Odds ratio (OR) and 95% confidence interval (95% CI) were estimated. The *F* values of all the SNPs were > 10, indicating a sufficient strength of the instrumental variables. The results from pleiotropy analysis indicated that most SNPs showed no horizontal pleiotropy (*p* > 0.05). The ferritin level had a causal effect on decreased eGFRcrea level in diabetes mellitus patients (OR = 0.937, 95% CI: 0.887–0.990) and increased risk of T1DM with renal complications (OR = 1.783, 95% CI: 1.005–3.162). TIBC level was causally associated with decreased risk of diabetic nephropathy (OR = 0.864, 95% CI: 0.771–0.968) and T1DM with renal complications (OR = 0.743, 95% CI: 0.603–0.916). Ferritin level had a causal effect on eGFRcrea level in diabetes mellitus patients and T1DM with renal complications. In conclusion, TIBC levels are causally linked to a lower risk of both diabetic nephropathy and T1DM with renal complications. The findings might provide a reference for using TIBC levels as a biomarker for prevention and treatment of diabetic nephropathy in the future. As the study was an MR analysis based on gene, the value of TIBC levels still should be validated in large prospective trials.

## 1. Introduction

Diabetic nephropathy is one of the most common and serious complications of diabetes mellitus, which is characterized by progressive impairment of the kidneys' delicate filtration units, known as nephrons, resulting from prolonged hyperglycemia [1, 2]. At present, it is projected that there will be approximately 552 million individuals worldwide with diabetic nephropathy by 2030, nearly 48% of whom will reside in China and India [3]. Diabetic nephropathy is a prominent etiology for the development of chronic kidney disease and end-stage renal disease (ESRD), which is a significant contributor to mortality among individuals with diabetes mellitus [4]. A prospective cohort study based on the Third National Health and Nutrition Examination Survey reported that the 10-year cumulative standardized mortality risk increased from 7.7% in individuals without diabetes mellitus or kidney disease to 11.5% in those with diabetes mellitus but no kidney disease and further rose to 31.1% in individuals with both diabetes mellitus and kidney disease. Among patients who progress to ESRD, the annualized mortality rate is approximately 20% [5]. Timely diagnosis and treatment of diabetic nephropathy can effectively halt the progressive decline in renal function among individuals with diabetes mellitus [6]. Biomarkers such as homeostasis of zinc [7], *β*-carotene, magnesium and zinc [8], and copper [9] were previously reported to be related to the progression of diabetic nephropathy. However, these findings were based on observational studies. Therefore, identifying more reliable biomarkers associated with the risk of diabetic nephropathy is of great value for the future management of patients with diabetes mellitus.

As one of the essential trace elements in human health, iron plays an important role in the process of iron overload [10]. Iron deficiency is associated with metabolic disorders, and iron homeostasis disturbance causes a variety of adverse effects [11, 12]. Observational studies have indicated dysregulated iron metabolism in individuals with diabetes mellitus. Several biomarkers of iron status, including serum iron and ferritin, have shown significant associations with renal function and the progression of diabetic nephropathy; for example, they revealed that ferritin levels were markedly lower in patients with nondiabetic kidney disease compared to those with diabetic kidney disease (117.00 *μ*g/L vs. 203.00 *μ*g/L) [13–15]. However, the results from these observational studies were not consistent, and the causal relationship between iron status and renal functions including diabetic nephropathy was still unclear.

Mendelian randomization (MR) approach represents a potential method for inferring causality, specifically designed to estimate the causal effects of exposure factors on outcomes [16]. The use of MR allows for the control of potential confounding factors and avoidance of biases caused by reverse causation, making it superior to traditional observational studies [17]. The reliability of the results from previous observational studies on the association between iron status and diabetic nephropathy was limited due to the confounding factors such as ferroptosis-related biomarkers, and various immune and inflammatory markers were not measured. Also, whether altered iron homeostasis was a consequence or a driver of disease progression in patients with diabetic nephropathy was unclear [13–15]. A previous MR study has explored the effect of serum iron on renal function [18]. Other MR studies also analyzed the causal association between iron homeostasis and the risk of diabetes mellitus [19, 20]. However, the evidence on the causal associations of iron homeostasis indicators with renal function and diabetic nephropathy in diabetic patients was still lacking.

In this study, MR analysis was used to clarify the causal effect of iron status on renal function and the risk of diabetic nephropathy in diabetic patients.

## 2. Methods

### 2.1. Study Design and Data Sources

**Figure 1:**
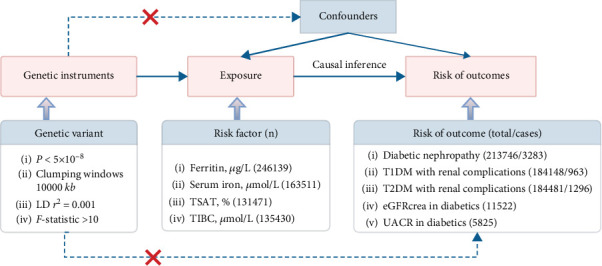
The directed acyclic graph of three assumptions of exposure and outcomes.

The present study was an MR analysis, which necessitates the examination of three assumptions to establish the appropriateness of single nucleotide polymorphisms (SNPs) as genetic instrumental variables for unbiasedly estimating causal impact. The first assumption was that the genetic instrumental variables would consistently and strongly correlate with the exposure. The second assumption was that these variables would remain independent of any confounding factors. The third assumption was that there would be no direct link between the genetic instrumental variables and the outcome; instead, their impact on the outcome should be mediated solely through their association with the exposure [21]. The directed acyclic graph of exposure and outcomes is exhibited in Figure 1. The data of exposures including ferritin, serum iron, transferrin saturation (TSAT), and total iron-binding capacity (TIBC) were obtained from a genome-wide association study (GWAS) yielding 46 new loci associated with biomarkers of iron homeostasis [22]. The outcomes were diabetic nephropathy, Type 1 diabetes mellitus (T1DM) with renal complications, Type 2 diabetes mellitus (T2DM) with renal complications, estimated glomerular filtration rate (creatinine) (eGFRcrea) in diabetes mellitus, and urinary albumin-to-creatinine ratio (UACR) in diabetes mellitus. The summary GWAS data of diabetic nephropathy, T1DM with renal complications, and T2DM with renal complications were obtained in FinnGen Biobank Analysis Round 5 (https://r5.finngen.fi/pheno). The FinnGen research project is a collaborative effort between the public and private sectors, integrating genotype data from Finnish biobanks with digital health record data from Finnish health registries. FinnGen offers a unique opportunity to investigate the relationship between genetic variation and disease trajectories in a genetically isolated population. As an expanding initiative, FinnGen is aimed at including 500,000 individuals by the end of 2023 [23]. The summary GWAS data of eGFRcrea in diabetics was extracted from a collaborative meta-analysis including nearly 62 studies with a distributive data model [24]. UACR in diabetes data was identified in a combined GWAS [25]. Since these databases were publicly available, the institutional review board of Shenzhen Third People's Hospital, The Second Affiliated Hospital, School of Medicine, Southern University of Science and Technology, has waived the ethical approval. All patients had provided informed consent in each involved GWAS. All methods were performed in accordance with the relevant guidelines and regulations.

### 2.2. SNP Selection

The data sources of SNPs related to ferritin, serum iron, TSAT, and TIBC are exhibited in Table 1. In total, 155,250 Icelanders were genotyped using Illumina SNP chips, and their genotypes were phased using long-range phasing. The GWAS from Denmark was performed using 19 million markers identified through whole-genome sequencing of 2816 Danes that were subsequently imputed into 84,386 chip-typed individuals. The GWAS from the United Kingdom was performed with 19 million markers from the UK10K and 1000 Genomes Phase 3 reference panel, imputed into 43,059 chip-typed individuals participating in the INTERVAL study. SNPs associated with exposures were selected with a threshold of *p* < 5 × 10^−8^. SNPs with genetic linkage disequilibrium (clump windows = 10,000 kb, *r*^2^ = 0.001) were excluded. Furthermore, palindrome SNPs were dropped from the analysis using a more conservative approach.

### 2.3. Outcome Data

The data of diabetic nephropathy, T1DM with renal complications, and T2DM with renal complications were obtained from 218,792 individuals of Finnish descent. The total samples and cases are displayed in Table 1. The eGFRcrea was estimated according to the four-variable Modification of Diet in Renal Disease (MDRD) Study Equation [26]. UACR was conducted in a combination of genome-wide association studies and independent replication in up to 5825 individuals of European ancestry with diabetes mellitus [25].

### 2.4. Statistical Analysis

The causal associations of ferritin, serum iron, TSAT and TIBC with diabetic nephropathy, T1DM with renal complications, T2DM with renal complications, eGFRcrea, or UACR were analyzed using MR analysis. The primary analytical method employed was the inverse variance-weighted (IVW) method, with support from the other methods including constrained maximum likelihood and model averaging (cML-MA), MR robust adjusted profile score (MR-RAPS), MR-Egger, simple mode, weighted median, weighted mode, MR-Pleiotropy Residual Sum and Outlier (MR-PRESSO) method, and radial MR. Detailed information on these methods is shown in Supporting Information. MR-Egger regression was applied for evaluating the potential pleiotropic effects of the SNPs using mr_pleiotropy_test() from TwoSampleMR. SNPs directly associated with diabetic nephropathy, T1DM with renal complications, T2DM with renal complications, eGFRcrea, or UACR, rather than through ferritin, serum iron, TSAT, and TIBC, were excluded. MR-Egger method is a causal hypothesis test that provides an estimation of causal impact when assuming all SNPs to be invalid [27]. The Cochrane's *Q* statistic was employed to assess the heterogeneity of SNPs, with a significance level set at *p* < 0.05 to indicate statistically significant heterogeneity. Leave-one-out analysis was utilized to find any individual SNP associated with exposures influencing outcomes. Odds ratio (OR) and 95% confidence interval (95% CI) were estimated. Statistical analysis was completed by R Version 4.2.3 (2023-03-15ucrt). MR analysis was performed using TwoSampleMR (Version 0.5.7), MRInstruments (Version 0.3.2), Mr. Raps (Version 0.2), MRcML (Version 0.0.0.9), MRPRESSO (Version 1.0), and RadialMR packages (Version 1.1).

## 3. Results

### 3.1. Selection of SNPs Related to Exposures

According to the data in Table 2, 42 SNPs related to ferritin, 16 SNPs related to serum iron, 16 SNPs associated with TIBC, and 12 SNPs associated with TSAT were identified according to the threshold of *p* < 5 × 10^−8^. After removing SNPs with genetic linkage disequilibrium, 31 SNPs associated with ferritin, 13 SNPs associated with serum iron, 13 SNPs associated with TIBC, and 9 SNPs associated with TSAT were included. Then, palindrome SNPs were omitted. Concerning diabetic nephropathy as the outcome, 30 SNPs associated with ferritin, 19 SNPs associated with serum iron, 9 SNPs associated with TIBC, and 9 SNPs associated with TSAT were finally included. Concerning eGFRcrea as the outcome, 19 SNPs associated with ferritin, 10 SNPs associated with serum iron, 9 SNPs associated with TIBC, and 8 SNPs associated with TSAT were finally included. Concerning T1DM with renal complications as the outcome, 30 SNPs associated with ferritin, 13 SNPs associated with serum iron, 13 SNPs associated with TIBC, and 9 SNPs associated with TSAT were finally included. Concerning T2DM with renal complications as the outcome, 30 SNPs associated with ferritin, 13 SNPs associated with serum iron, 9 SNPs associated with TIBC, and 8 SNPs associated with TSAT were finally included. Concerning UACR as the outcome, 20 SNPs associated with ferritin, 11 SNPs associated with serum iron, 10 SNPs associated with TIBC, and 9 SNPs associated with TSAT were finally included. The *F* values of all the SNPs were > 10, indicating a sufficient strength of the instrumental variables.

### 3.2. The Pleiotropy Analysis and Heterogeneity Test of the SNPs Related to Exposures

The results from pleiotropy analysis indicated that most SNPs showed no horizontal pleiotropy (*p* > 0.05). SNPs related to serum iron with eGFRcrea as the outcome, SNPs related to TSAT with diabetic nephropathy as the outcome, and T2DM with renal complications as the outcome had potential horizontal pleiotropy. The results from the heterogeneity test revealed that SNPs related to TSAT with T2DM with renal complications as the outcome showed heterogeneity, and radial MR identified that rs7385804 was the outlier SNP (Table 3).

### 3.3. MR Analysis on the Associations of Iron Status With the Renal Function and Diabetic Nephropathy in Patients With Diabetes Mellitus

**Figure 2:**
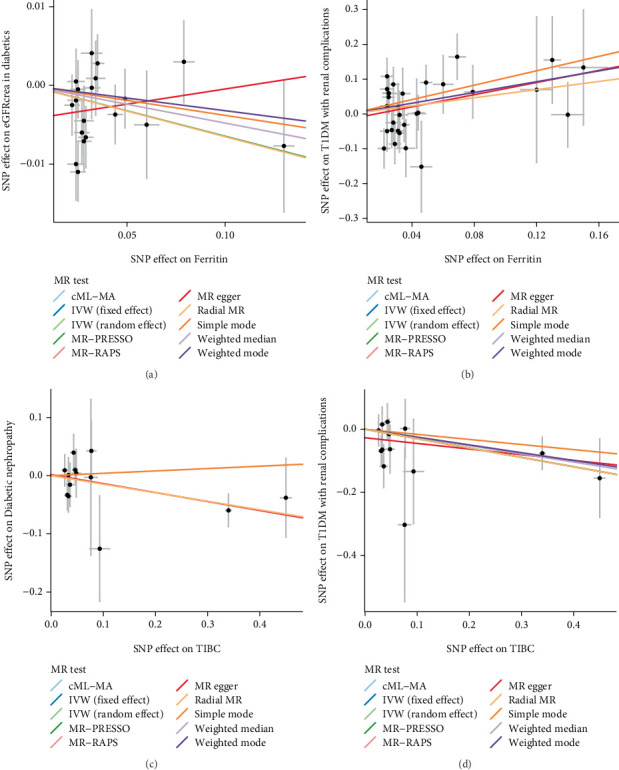
The scatter plot showing the causal effects of ferritin on eGFRcrea level and T1DM with renal complications, as well as the causal effects of TIBC level on diabetic nephropathy and T1DM with renal complications.

We found that ferritin level had a causal effect on decreased eGFRcrea level in diabetes mellitus patients (OR = 0.937, 95% CI: 0.887–0.990) and increased risk of T1DM with renal complications (OR = 1.783, 95% CI: 1.005–3.162). TIBC level was causally associated with decreased risk of diabetic nephropathy (OR = 0.864, 95% CI: 0.771–0.968) and T1DM with renal complications (OR = 0.743, 95% CI: 0.603–0.916) (Table 4). The scatter plot interpreted the validity of the standard IVW estimate and pleiotropy robust methods, indicating the causal effects of ferritin on eGFRcrea level and T1DM with renal complications, as well as the causal effects of TIBC level on diabetic nephropathy and T1DM with renal complications, were significant (Figure 2). The IVW radial plot showing the causal association between ferritin and eGFRcrea level, ferritin and T1DM with renal complications, TIBC and diabetic nephropathy, and TIBC and T1DM with renal complications were presented in Figures S1–S4, respectively. The causal relationship of iron status with the renal function and diabetic nephropathy in patients with diabetes mellitus was also evaluated via other MR methods, and the results are exhibited in Table S1.

### 3.4. Sensitivity Analysis

**Figure 3:**
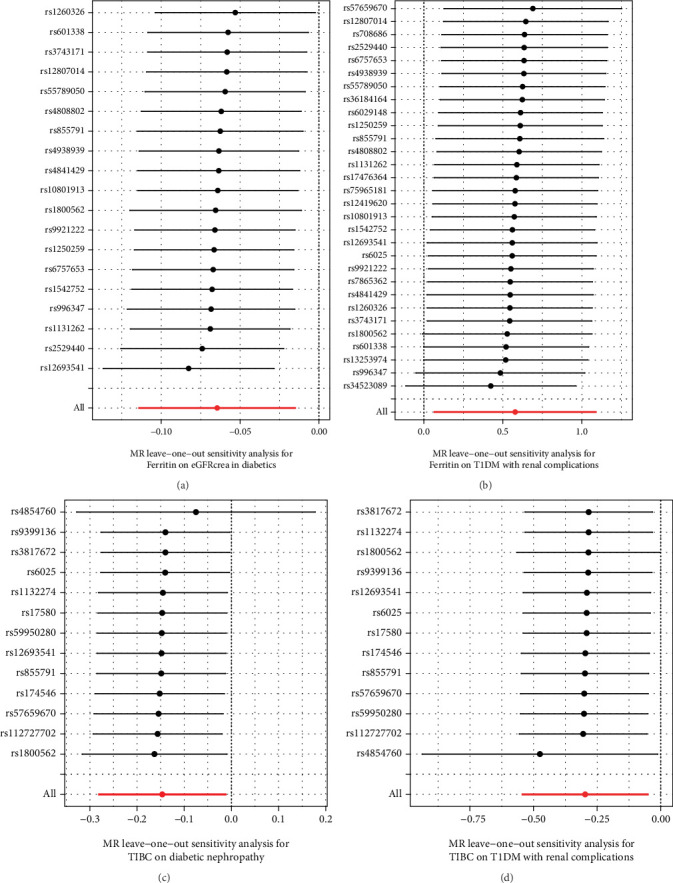
The leave-one-out analysis exploring individual SNP skewing the causal inferences of ferritin on eGFRcrea level and T1DM with renal complications, as well as the causal effects of TIBC level on diabetic nephropathy and T1DM with renal complications.

The results of leave-one-out analysis demonstrated that no individual SNP skewed the causal inferences of ferritin on eGFRcrea level and T1DM with renal complications, as well as the causal effects of TIBC level on diabetic nephropathy and T1DM with renal complications (Figure 3).

## 4. Discussion

In the present study, the causal effect of iron status on renal function and the risk of diabetic nephropathy in diabetic patients were evaluated using MR analysis. The results depicted that ferritin level was causally associated with decreased eGFRcrea level in diabetes mellitus patients and increased risk of T1DM with renal complications. TIBC level was causally associated with decreased risk of diabetic nephropathy and T1DM with renal complications. The findings hold promise for providing a reference for identifying interventions on the management of the renal function in patients with diabetes mellitus.

Previously, evidence revealed that several trace element–related biomarkers were associated with the pathogenesis and progression of diabetic nephropathy. Qin et al. indicated that zinc might regulate the hypoxia-inducible factor-1*α* in diabetic kidney disease and modulate the development of the disease [7]. Over a 3-month period, combination therapy of *β*-carotene, magnesium, and zinc with metformin demonstrated superior glycemic control, significant protection against potential neuropathy and sensorimotor dysfunction, preserved retinal integrity, and notable management of diabetic nephropathy when compared to metformin monotherapy [8]. Ming et al. demonstrated that copper-related genes might be novel diagnostic biomarkers for the theoretical foundation and treatment targets in diabetic nephropathy [9]. In addition, the iron metabolism levels in diabetic patients commonly exhibit abnormalities [15]. The progression of diabetic nephropathy is characterized by the development of renal fibrosis, resulting in significant disruption to kidney structure and function. There is a positive correlation between the extent of renal cortical interstitial fibrosis and serum creatinine levels at the time of biopsy in patients with diabetic nephropathy [28]. Previously, there was evidence indicated that the levels of serum creatinine and eGFR showed a significant correlation with the iron profile in patients with chronic kidney disease [29]. Another study revealed that patients with chronic kidney disease are often iron deficient, even when not anemic [30]. Praveen et al. found that TIBC and serum iron were significantly decreased in patients with total iron deficiency, and serum ferritin and iron were reduced in T2DM patients with anemia [31]. On the other hand, excess iron accumulation was reported to mediate senescence in diabetic kidney injury [32]. The excessive accumulation of iron can generate a significant amount of free radicals, leading to substantial cellular and tissue damage as well as the induction of fibrosis, and the surplus iron may trigger signals that promote fibrosis, accelerating the onset of illness and exacerbating kidney pathology [33]. In our study, there were causal associations of ferritin with eGFRcrea and T1DM with renal complications in diabetes mellitus patients. We also found the causal associations of TIBC with diabetic nephropathy and T1DM with renal complications. Notably, only ferritin and TIBC exhibited significant causal associations with specific renal outcomes, whereas serum iron and TSAT did not. These findings might be due to the following factors. Biologically, ferritin serves as a marker of total body iron stores and is also influenced by inflammatory processes, while TIBC reflects transferrin availability and iron-binding capacity [34]. These biomarkers may better capture chronic alterations in iron metabolism relevant to renal dysfunction, compared to serum iron and TSAT, which are more susceptible to short-term physiological fluctuations and diurnal variation. Methodologically, differences in the number and strength of instrumental variables (SNPs) across biomarkers likely contributed to variability in statistical power. Ferritin and TIBC were supported by a greater number of robust instruments, improving the precision of their causal estimates. In contrast, limited or weaker instruments for serum iron and TSAT may have constrained the ability to detect associations. Furthermore, it is possible that specific iron pathways, such as iron storage (ferritin) and transport capacity (TIBC), are more directly implicated in the pathophysiology of diabetic nephropathy than other iron metrics. Residual pleiotropy, despite sensitivity analyses including MR-Egger and MR-PRESSO, may have influenced the null findings.

The potential mechanisms might be that elevated iron levels have the potential to impair the functionality of pancreatic *β* cells [35]. Also, elevated iron status might reduce the insulin sensitivity and adiponectin secretion in adipocytes [36]. Another study suggested that excessive dietary iron intake may enhance the activity of adenosine monophosphate–activated protein kinase C in both the liver and skeletal muscle, while also impairing insulin signaling pathways in mice [37]. On the contrary, there was also evidence indicated that high iron levels could deteriorate the development of T2DM via regulating liver glycogen deposition, blood glucose, and insulin levels in db/db mice [38]. Insufficient iron levels can lead to oxidative stress, specifically causing mitochondrial dysfunction as a result of oxidative stress–related damage to mitochondrial DNA [39, 40].

In the current study, MR analysis was applied to analyze the causal effect of iron status on renal function and the risk of diabetic nephropathy in diabetic patients. The strength of this study lies in the study design. Two-sample MR was based on gene-level biomarkers and can maximize the control of the common confounding in traditional epidemiology, overcoming the limitation of uncontrolled confounding factors in traditional observational studies. Also, MR analysis can avoid the biases caused by reverse causation and can obtain the conclusion that the altered iron homeostasis was a driver of disease progression in patients with diabetic nephropathy. The findings of our study may hold practical significance at both the clinical and public health levels.

At the clinical level, elucidating the precise role of iron metabolism in regulating diabetic nephropathy is of critical importance, particularly as iron is increasingly perceived as potentially harmful. Some researchers have even proposed the use of iron chelators for the prevention and management of kidney disease [41].

Iron metabolism within the kidney involves highly complex physiological processes, and there may exist a narrow range of optimal iron levels. This phenomenon could account for the findings of “inverse U-shaped” association between iron-related biomarkers and risk of diabetes renal dysfunction. In addition, at the public health level, policies such as iron supplementation should be proposed for those with nutritional deficiency, particularly in women and children. The results might help make some targets for clinical prevention or intervention of patients with diabetic patients.

Some limitations existed in our study. Firstly, the data source of this study was from European descent, which might reduce the bias caused by different races, but the generalization to other races should be conducted with caution. Secondly, there was no individual data for further exploration of the nonlinear correlation between iron status and renal function in patients with diabetes mellitus. Thirdly, both IVs associated with exposure and outcome were relatively small, and the results need to be verified in larger samples and other ethnic populations.

## 5. Conclusions

This study evaluated the causal association between iron status and the risk of renal function and diabetic nephropathy. The results demonstrated that ferritin level was causally associated with decreased eGFRcrea level and increased risk of T1DM with renal complications in diabetes mellitus patients. TIBC level was causally associated with decreased risk of diabetic nephropathy and T1DM with renal complications. The findings suggested the potential effect of iron levels on kidney function in the diabetes mellitus patients. Further research is required to confirm this causal association, and the underlying mechanisms still need investigation.

## Figures and Tables

**Table 1:** The information of biomarkers of iron status and renal functions.

**Type**	**Phenotype (unit)**	**Total sample size (case)**	**GWAS ID**	**PMID/consortium**
Exposures	Ferritin (*μ*g/L)	246,139	GCST011369	33536631
	Serum iron (*μ*mol/L)	163,511	GCST011367	33536631
	TSAT (%)	131,471	GCST011366	33536631
	TIBC (*μ*mol/L)	135,430	GCST011368	33536631

Outcomes	Diabetic nephropathy	213,746 (3283)	finn-b DM_NEPHROPATHY	FinnGen
	T1DM with renal complications	184,148 (963)	finn-b-E4_DM1REN	FinnGen
	T2DM with renal complications	184,481 (1296)	finn-b-E4_DM2REN	FinnGen
	EGFRcrea in diabetics (mL min^−1^ per 1.73 m^2^)	11,522	ebi-a-GCST003373	26831199
	UACR in diabetics (mg/g)	5825	ieu-a-1100	CKDGen/26631737

Abbreviations: eGFRcrea, estimated glomerular filtration rate by serum creatinine; GWAS, genome-wide association studies; T1DM, Type 1 diabetes mellitus; T2DM, Type 2 diabetes mellitus; TIBC, total iron-binding capacity; TSAT, transferrin saturation; UACR, urinary albumin-to-creatinine ratio.

**Table 2:** The selection procedure of SNPs associated with biomarkers of iron status.

**Exposure**	**Outcome**	**Selected SNP (** **p** < 5 × 10^−8^**)**	**Omitting SNPs with LD**	**Dropping palindromic SNPs**	**F** ** statistic**	**R** ^2^
Ferritin	Diabetic nephropathy	42	31	30	103	0.013
	eGFRcrea in diabetics	42	31	19	91	0.007
	T1DM with renal complications	42	31	30	103	0.013
	T2DM with renal complications	42	31	30	103	0.013
	UACR in diabetics	42	31	20	112	0.009

Serum iron	Diabetic nephropathy	16	13	13	205	0.016
	eGFRcrea in diabetics	16	13	10	255	0.016
	T1DM with renal complications	16	13	13	205	0.016
	T2DM with renal complications	16	13	13	205	0.016
	UACR in diabetics	16	13	11	235	0.016

TIBC	Diabetic nephropathy	16	13	13	182	0.017
	eGFRcrea in diabetics	16	13	9	242	0.016
	T1DM with renal complications	16	13	13	182	0.017
	T2DM with renal complications	16	13	13	182	0.017
	UACR in diabetics	16	13	10	225	0.017

TSAT	Diabetic nephropathy	12	9	9	302	0.021
	eGFRcrea in diabetics	12	9	8	334	0.020
	T1DM with renal complications	12	9	9	302	0.021
	T2DM with renal complications	12	9	9	302	0.021
	UACR in diabetics	12	9	9	302	0.021

Abbreviations: eGFRcrea, estimated glomerular filtration rate by serum creatinine; LD, linkage disequilibrium; SNP, single nucleotide polymorphism; T1DM, Type 1 diabetes mellitus; T2DM, Type 2 diabetes mellitus; TIBC, total iron-binding capacity; TSAT, transferrin saturation; UACR, urinary albumin-to-creatinine ratio.

**Table 3:** The pleiotropic and heterogeneity test of SNPs associated with iron status.

**Exposure**	**Outcome**	**Heterogeneity test**				**Horizontal pleiotropic test**				**Radial MR outlier SNP**
		**MR-Egger ** **Q**	**p**	**IVW ** **Q**	**p**	**Egger intercept**	**p**	**MR-PRESSO global test**	**p**	
Ferritin	Diabetic nephropathy	−0.003	0.850	34.12	0.197	34.17	0.233	35.76	0.268	—
	eGFRcrea in diabetics	−0.004	0.073	17.91	0.395	21.76	0.243	24.02	0.258	—
	T1DM with renal complications	−0.015	0.554	35.34	0.160	35.79	0.180	38.21	0.194	—
	T2DM with renal complications	−0.019	0.330	23.36	0.715	24.34	0.712	25.52	0.736	—
	UACR in diabetics	0.004	0.826	25.92	0.102	25.99	0.130	29.63	0.116	—

Serum iron	Diabetic nephropathy	−0.017	0.229	12.48	0.328	14.32	0.281	16.62	0.326	—
	eGFRcrea in diabetics	0.005	0.035	3.64	0.888	10.04	0.347	12.20	0.415	—
	T1DM with renal complications	−0.031	0.214	11.75	0.383	13.61	0.327	16.00	0.374	—
	T2DM with renal complications	−0.037	0.088	10.90	0.452	14.40	0.276	17.37	0.308	—
	UACR in diabetics	−0.008	0.594	6.30	0.710	6.60	0.762	12.32	0.589	—

TIBC	Diabetic nephropathy	0.002	0.893	8.43	0.674	8.45	0.749	10.05	0.794	—
	eGFRcrea in diabetics	0.002	0.333	4.57	0.713	5.65	0.687	9.35	0.656	—
	T1DM with renal complications	−0.027	0.235	6.88	0.809	8.46	0.749	11.08	0.751	—
	T2DM with renal complications	0.020	0.298	10.41	0.494	11.61	0.478	12.52	0.608	—
	UACR in diabetics	0.023	0.172	9.31	0.317	11.94	0.217	15.10	0.427	—

TSAT	Diabetic nephropathy	−0.006	0.829	15.17	0.034	15.27	0.054	19.51	0.118	—
	eGFRcrea in diabetics	0.004	0.109	0.99	0.986	4.54	0.716	5.32	0.782	—
	T1DM with renal complications	−0.010	0.825	13.20	0.067	13.30	0.102	16.08	0.175	—
	T2DM with renal complications	−0.036	0.387	15.13	0.034	16.97	0.030	21.93	0.080	rs7385804
	UACR in diabetics	0.009	0.647	1.69	0.975	1.92	0.984	2.54	0.982	—

Abbreviations: eGFRcrea, estimated glomerular filtration rate by serum creatinine; IVW, inverse variance-weighted; LD, linkage disequilibrium; MR, Mendelian randomization; SNP, single nucleotide polymorphism; T1DM, Type 1 diabetes mellitus; T2DM, Type 2 diabetes mellitus; TIBC, total iron-binding capacity; TSAT, transferrin saturation; UACR, urinary albumin-to-creatinine ratio.

**Table 4:** The IVW MR results of the causal association between iron status and renal function.

**Exposure**	**Outcome**	**nSNPs**	**IVW (random effect)**		**IVW (fixed effect)**	
			**OR (95% CI)**	**p**	**OR (95% CI)**	**p**
Ferritin	Diabetic nephropathy	30	0.943 (0.694–1.281)	0.706	0.943 (0.711–1.250)	0.683
	eGFRcrea in diabetics	19	0.937 (0.887–0.990)	0.021	0.937 (0.892–0.986)	0.011
	T1DM with renal complications	30	1.783 (1.005–3.162)	0.048	1.783 (1.064–2.986)	0.028
	T2DM with renal complications	30	0.864 (0.572–1.307)	0.490	0.864 (0.550–1.358)	0.527
	UACR in diabetics	20	1.236 (0.789–1.934)	0.355	1.236 (0.842–1.813)	0.279

Serum iron	Diabetic nephropathy	13	0.897 (0.702–1.147)	0.388	0.897 (0.717–1.124)	0.345
	eGFRcrea in diabetics	10	0.993 (0.961–1.026)	0.665	0.993 (0.963–1.024)	0.647
	T1DM with renal complications	13	0.980 (0.633–1.519)	0.929	0.980 (0.650–1.479)	0.924
	T2DM with renal complications	13	0.720 (0.486–1.067)	0.102	0.720 (0.503–1.031)	0.073
	UACR in diabetics	11	1.013 (0.826–1.242)	0.902	1.013 (0.788–1.301)	0.920

TIBC	Diabetic nephropathy	13	0.864 (0.771–0.968)	0.012	0.864 (0.754–0.990)	0.035
	eGFRcrea in diabetics	9	1.008 (0.993–1.024)	0.274	1.008 (0.990–1.027)	0.358
	T1DM with renal complications	13	0.743 (0.603–0.916)	0.005	0.743 (0.579–0.954)	0.020
	T2DM with renal complications	13	0.926 (0.749–1.146)	0.482	0.926 (0.746–1.150)	0.489
	UACR in diabetics	10	0.918 (0.772–1.091)	0.330	0.918 (0.790–1.066)	0.262

TSAT	Diabetic nephropathy	9	1.093 (0.828–1.443)	0.531	1.093 (0.894–1.336)	0.386
	eGFRcrea in diabetics	8	0.988 (0.967–1.009)	0.269	0.988 (0.962–1.015)	0.373
	T1DM with renal complications	9	1.207 (0.751–1.939)	0.437	1.207 (0.836–1.743)	0.316
	T2DM with renal complications	9	0.928 (0.582–1.480)	0.754	0.928 (0.674–1.278)	0.647
	UACR in diabetics	9	0.933 (0.840–1.038)	0.202	0.933 (0.752–1.159)	0.532

Abbreviations: CI, confidence interval; eGFRcrea, estimated glomerular filtration rate by serum creatinine; IVW, inverse variance-weighted; MR, Mendelian randomization; OR, odds ratio; SNP, single nucleotide polymorphism; T1DM, Type 1 diabetes mellitus; T2DM, Type 2 diabetes mellitus; TIBC, total iron-binding capacity; TSAT, transferrin saturation; UACR, urinary albumin-to-creatinine ratio.

## Data Availability

The data that support the findings of this study are openly available in GWASs at https://gwas.mrcieu.ac.uk/datasets/ukb-d-ASTHMA_CHILD/.

## Supporting Information

Supporting Information 1 Figure S1: The IVW radial plot showing the causal association between ferritin and eGFRcrea level.

Supporting Information 2 Figure S2: The IVW radial plot showing the causal association between ferritin and T1DM with renal complications.

Supporting Information 3 Figure S3: The IVW radial plot showing the causal association between TIBC and diabetic nephropathy.

Supporting Information 4 Figure S4: The IVW radial plot showing the causal association between TIBC and T1DM with renal complications.

Supporting Information 5 Table S1: The MR estimations of a causal association between iron status and renal function using other MR methods.

## References

[1] Valencia W. M., Florez H. (2017). How to Prevent the Microvascular Complications of Type 2 Diabetes Beyond Glucose Control. *BMJ*.

[2] Qi C., Mao X., Zhang Z., Wu H. (2017). Classification and Differential Diagnosis of Diabetic Nephropathy. *Journal of Diabetes Research*.

[3] Wheeler D. C., Stefánsson B. V., Jongs N. (2021). Effects of Dapagliflozin on Major Adverse Kidney and Cardiovascular Events in Patients With Diabetic and Non-Diabetic Chronic Kidney Disease: A Prespecified Analysis From the DAPA-CKD Trial. *Lancet Diabetes & Endocrinology*.

[4] Liu J., Liu Z., Sun W. (2023). Role of Sex Hormones in Diabetic Nephropathy. *Frontiers in Endocrinology*.

[5] Griffin T. P., O’Shea P. M., Smyth A. (2021). Burden of Chronic Kidney Disease and Rapid Decline in Renal Function Among Adults Attending a Hospital-Based Diabetes Center in Northern Europe. *BMJ Open Diabetes Research & Care*.

[6] Kushner P., Peach E., Wittbrodt E. (2022). Investigating the Global Prevalence and Consequences of Undiagnosed Stage 3 Chronic Kidney Disease: Methods and Rationale for the REVEAL-CKD Study. *Clinical Kidney Journal*.

[7] Qin W., Nie P., Hui X. (2025). Research Progress of Hypoxia-Inducible Factor-1*α* and Zinc in the Mechanism of Diabetic Kidney Disease. *Frontiers in Pharmacology*.

[8] Kateel R., Kashyap N. N., Reddy S. K. (2025). Chronic *β*-Carotene, Magnesium, and Zinc Supplementation Together With Metformin Attenuates Diabetes-Related Complications in Aged Rats. *Clinical Nutrition*.

[9] Ming J., Sana S., Deng X. (2022). Identification of Copper-Related Biomarkers and Potential Molecule Mechanism in Diabetic Nephropathy. *Frontiers in Endocrinology*.

[10] Von Holle A. (2024). Assessment of Iron Status. *Current Opinion in Clinical Nutrition and Metabolic Care*.

[11] Muckenthaler M. U., Rivella S., Hentze M. W., Galy B. (2017). A Red Carpet for Iron Metabolism. *Cell*.

[12] Pilling L. C., Tamosauskaite J., Jones G. (2019). Common Conditions Associated With Hereditary Haemochromatosis Genetic Variants: Cohort Study in UK Biobank. *BMJ*.

[13] Zhao L., Zou Y., Zhang J. (2020). Serum Transferrin Predicts End-Stage Renal Disease in Type 2 Diabetes Mellitus Patients. *International Journal of Medical Sciences*.

[14] Wu Y., Sun Y., Wu Y., Zhang K., Chen Y. (2023). Predictive Value of Ferroptosis-Related Biomarkers for Diabetic Kidney Disease: A Prospective Observational Study. *Acta Diabetologica*.

[15] Altamura S., Kopf S., Schmidt J. (2017). Uncoupled Iron Homeostasis in Type 2 Diabetes Mellitus. *Journal of Molecular Medicine*.

[16] Fang X., Deng Q., Yang H. (2024). Causal Association of Immune Cells and Endometriosis: A Mendelian Randomization Study. *Frontiers in Endocrinology*.

[17] Emdin C. A., Khera A. V., Kathiresan S. (2017). Mendelian Randomization. *Jama*.

[18] Del Greco M. F., Foco L., Pichler I. (2017). Serum Iron Level and Kidney Function: A Mendelian Randomization Study. *Nephrology Dialysis Transplantation*.

[19] Liang Y., Luo S., Wong T. H. T., He B., Schooling C. M., Au Yeung S. L. (2023). Association of Iron Homeostasis Biomarkers in Type 2 Diabetes and Glycaemic Traits: A Bidirectional Two-Sample Mendelian Randomization Study. *International Journal of Epidemiology*.

[20] Wang X., Fang X., Zheng W. (2021). Genetic Support of a Causal Relationship Between Iron Status and Type 2 Diabetes: A Mendelian Randomization Study. *Journal of Clinical Endocrinology & Metabolism*.

[21] Yang S., Wang X., Li Y., Zhou L., Guo G., Wu M. (2024). The Association Between Telomere Length and Blood Lipids: A Bidirectional Two-Sample Mendelian Randomization Study. *Frontiers in Endocrinology*.

[22] Bell S., Rigas A. S., Magnusson M. K. (2021). A Genome-Wide Meta-Analysis Yields 46 New Loci Associating With Biomarkers of Iron Homeostasis. *Communications Biology*.

[23] Yuan S., Carter P., Mason A. M., Burgess S., Larsson S. C. (2021). Coffee Consumption and Cardiovascular Diseases: A Mendelian Randomization Study. *Nutrients*.

[24] Pattaro C., Teumer A., Gorski M. (2016). Genetic Associations at 53 Loci Highlight Cell Types and Biological Pathways Relevant for Kidney Function. *Nature Communications*.

[25] Teumer A., Tin A., Sorice R. (2016). Genome-Wide Association Studies Identify Genetic Loci Associated With Albuminuria in Diabetes. *Diabetes*.

[26] Reynolds T. M., Twomey P. J. (2007). Implications of Method Specific Creatinine Adjustments on General Medical Services Chronic Kidney Disease Classification. *Journal of Clinical Pathology*.

[27] Bowden J., Davey Smith G., Burgess S. (2015). Mendelian Randomization With Invalid Instruments: Effect Estimation and Bias Detection Through Egger Regression. *International Journal of Epidemiology*.

[28] Zeng L. F., Xiao Y., Sun L. (2019). A Glimpse of the Mechanisms Related to Renal Fibrosis in Diabetic Nephropathy. *Advances in Experimental Medicine and Biology*.

[29] Shreewastav R. K., Joshi B. R., Yadav R., Katwal A., Shrestha S. (2023). Iron Profile and Status of Anemia With the Associated Factors in Chronic Kidney Disease Patients. *Journal of Nepal Health Research Council*.

[30] Greenwood S. A., Oliveira B. A., Asgari E. (2023). A Randomized Trial of Intravenous Iron Supplementation and Exercise on Exercise Capacity in Iron-Deficient Nonanemic Patients With CKD. *Kidney International Reports*.

[31] Praveen M., Jain N., Raizada N., Sharma S., Narang S., Madhu S. V. (2020). Anaemia in Patients With Type 2 Diabetes Mellitus Without Nephropathy Is Related to Iron Deficiency. *Diabetes & Metabolic Syndrome*.

[32] Cheng X., Li Y., Chen L. (2024). Excess Iron Accumulation Mediated Senescence in Diabetic Kidney Injury. *Journal of Biochemical and Molecular Toxicology*.

[33] Mehta K. J., Farnaud S. J., Sharp P. A. (2019). Iron and Liver Fibrosis: Mechanistic and Clinical Aspects. *World Journal of Gastroenterology*.

[34] Pandur E., Varga E., Tamási K., Pap R., Nagy J., Sipos K. (2019). Effect of Inflammatory Mediators Lipopolysaccharide and Lipoteichoic Acid on Iron Metabolism of Differentiated SH-SY5Y Cells Alters in the Presence of BV-2 Microglia. *International Journal of Molecular Sciences*.

[35] Cheng K., Ho K., Stokes R. (2010). Hypoxia-Inducible Factor-1alpha Regulates Beta Cell Function in Mouse and Human Islets. *Journal of Clinical Investigation*.

[36] Huang J., Simcox J., Mitchell T. C. (2013). Iron Regulates Glucose Homeostasis in Liver and Muscle via AMP-Activated Protein Kinase in Mice. *FASEB Journal*.

[37] Ma W., Feng Y., Jia L. (2019). Dietary Iron Modulates Glucose and Lipid Homeostasis in Diabetic Mice. *Biological Trace Element Research*.

[38] Walter P. B., Knutson M. D., Paler-Martinez A. (2002). Iron Deficiency and Iron Excess Damage Mitochondria and Mitochondrial DNA in Rats. *Proceedings of the National Academy of Sciences of the United States of America*.

[39] Knutson M. D., Walter P. B., Ames B. N., Viteri F. E. (2000). Both Iron Deficiency and Daily Iron Supplements Increase Lipid Peroxidation in Rats. *Journal of Nutrition*.

[40] Shah S. V., Rajapurkar M. M. (2009). The Role of Labile Iron in Kidney Disease and Treatment With Chelation. *Hemoglobin*.

[41] Gabrielsen J. S., Gao Y., Simcox J. A. (2012). Adipocyte Iron Regulates Adiponectin and Insulin Sensitivity. *Journal of Clinical Investigation*.

