# Supplementary figures and images for: Causal Associations of Iron Status With the Renal Function and Diabetic Nephropathy in Patients With Diabetes Mellitus: A Two-Sample Mendelian Randomization Study
Source: J Diabetes Res. 2025 Jul 30;2025:6658794. doi: 10.1155/jdr/6658794 (PMC12401605; doi:10.1155/jdr/6658794)

# IVW Radial (Ferritin on eGFRcrea in diabetics)

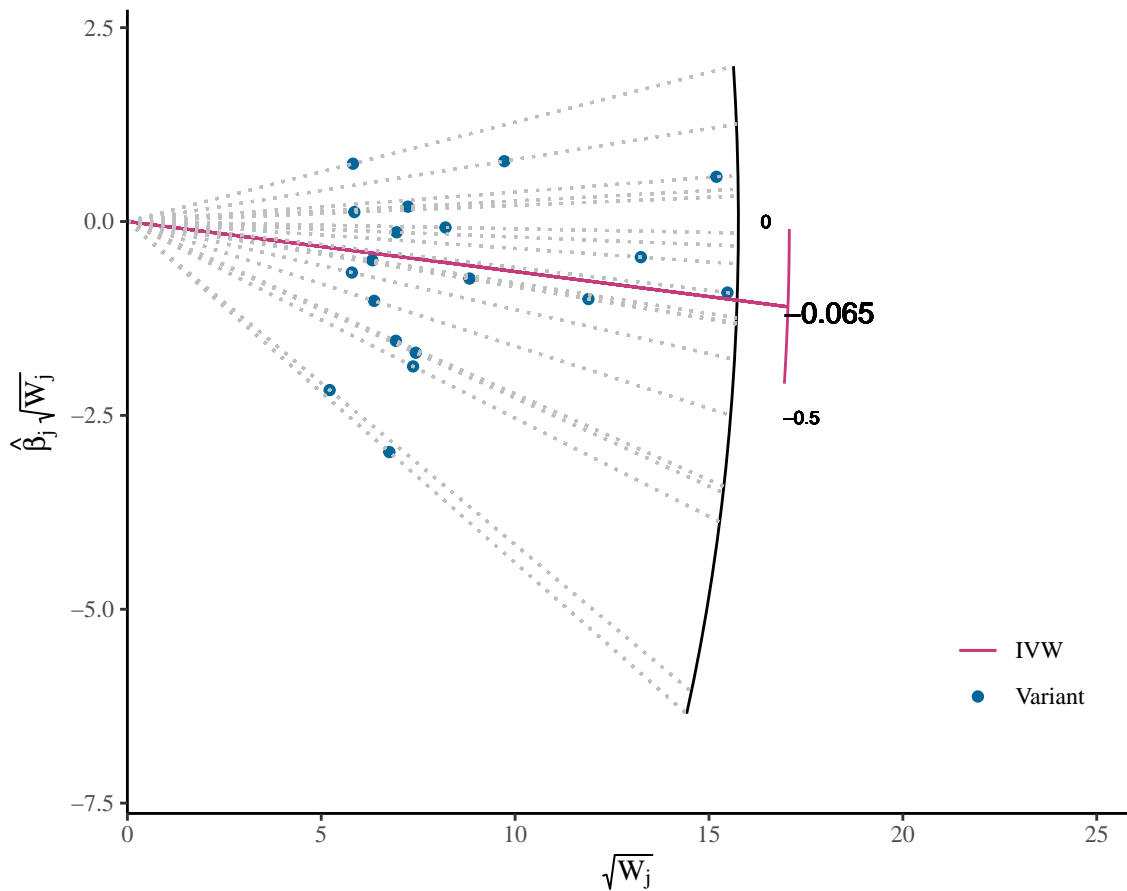

Supplement: Supporting Information 1 — Figure S1: The IVW radial plot showing the causal association between ferritin and eGFRcrea level. [file 6658794.f1.pdf]

# IVW Radial (Ferritin on T1DM with renal complications)

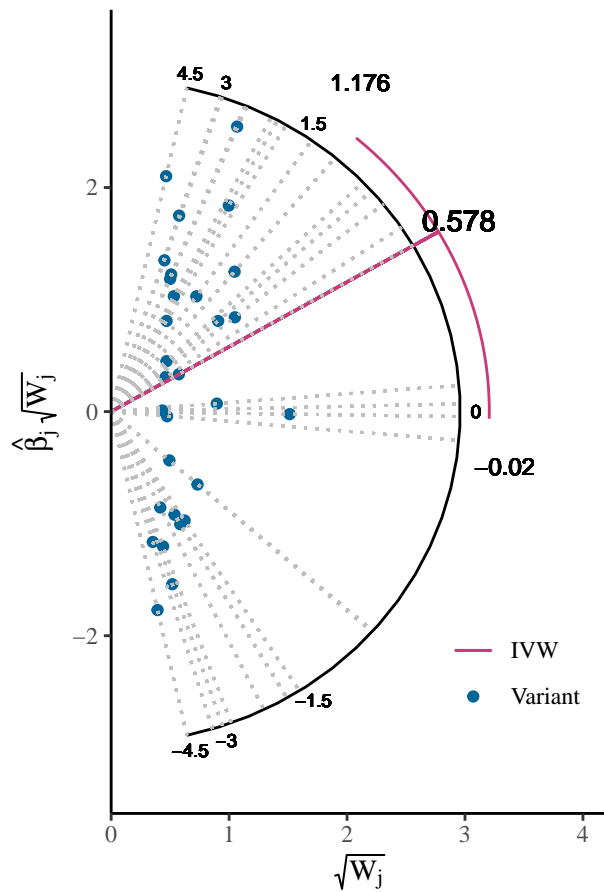

Supplement: Supporting Information 2 — Figure S2: The IVW radial plot showing the causal association between ferritin and T1DM with renal complications. [file 6658794.f2.pdf]

# IVW Radial (TIBC on diabetic nephropathy)

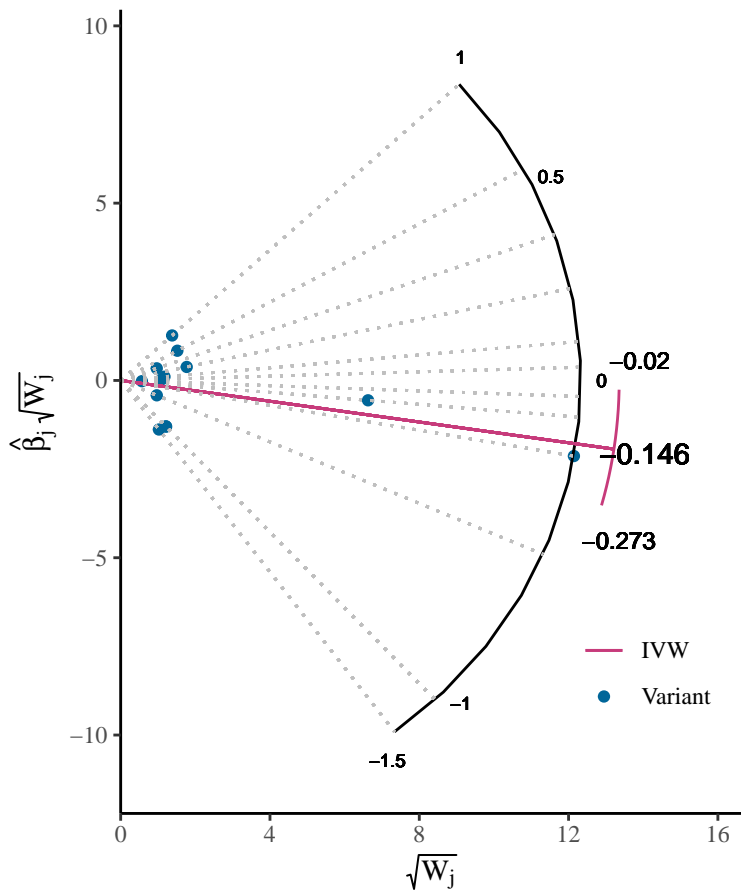

Supplement: Supporting Information 3 — Figure S3: The IVW radial plot showing the causal association between TIBC and diabetic nephropathy. [file 6658794.f3.pdf]

# IVW Radial (TIBC on T1DM with renal complications)

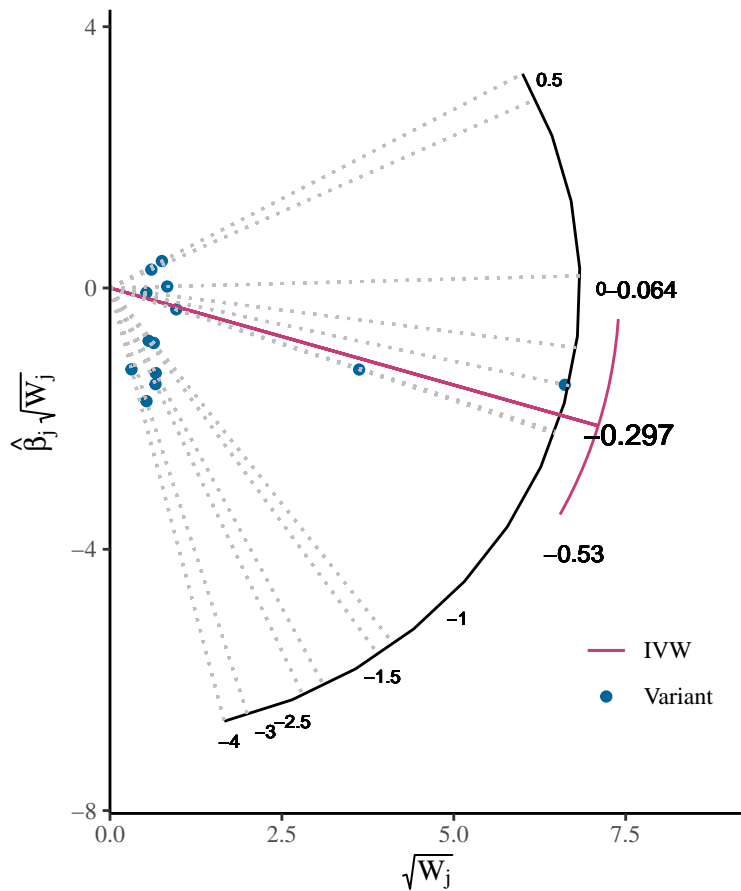

Supplement: Supporting Information 4 — Figure S4: The IVW radial plot showing the causal association between TIBC and T1DM with renal complications. [file 6658794.f4.pdf]
