# Supplementary material for: Causal Associations of Iron Status With the Renal Function and Diabetic Nephropathy in Patients With Diabetes Mellitus: A Two-Sample Mendelian Randomization Study
Source: J Diabetes Res. 2025 Jul 30;2025:6658794. doi: 10.1155/jdr/6658794 (PMC12401605; doi:10.1155/jdr/6658794)
Supplement: Supporting Information 5 — Table S1: The MR estimations of a causal association between iron status and renal function using other MR methods. [file 6658794.f5.pdf]

Supplementary Table 1 The MR estimations of causal association between iron status and renal function using other MR methods

	Outcome	Method	nSNPs	*OR* (95%*CI*)	*P*
Ferritin	Diabetic nephropathy	cML-MA	30	0.940 (0.707-1.250)	0.671
		MR-RAPS	30	0.942 (0.708-1.253)	0.682
		MR Egger	30	0.992 (0.540-1.823)	0.979
		Simple mode	30	0.824 (0.435-1.564)	0.559
		Weighted median	30	0.942 (0.636-1.394)	0.764
		Weighted mode	30	0.874 (0.565-1.352)	0.550
		MR-PRESSO	30	0.943 (0.694-1.281)	0.709
		Radial MR	30	0.942 (0.727-1.221)	0.656
	eGFRcrea in diabetics	cML-MA	19	0.937 (0.891-0.986)	0.012
		MR-RAPS	19	0.937 (0.891-0.985)	0.011
		MR Egger	19	1.039 (0.924-1.169)	0.529
		Simple mode	19	0.963 (0.861-1.076)	0.511
		Weighted median	19	0.953 (0.886-1.025)	0.198
		Weighted mode	19	0.968 (0.888-1.056)	0.479
		MR-PRESSO	19	0.937 (0.887-0.990)	0.033
		Radial MR	19	0.937 (0.888-0.988)	0.028
	T1D with renal complications	cML-MA	30	1.792 (1.064-3.019)	0.028
		MR-RAPS	30	1.796 (1.066-3.027)	0.028
		MR Egger	30	2.398 (0.774-7.428)	0.141
		Simple mode	30	2.816 (0.644-12.312)	0.180
		Weighted median	30	2.166 (0.982-4.778)	0.056
		Weighted mode	30	2.168 (0.791-5.943)	0.143
		MR-PRESSO	30	1.783 (1.005-3.162)	0.058
		Radial MR	30	1.794 (1.040-3.095)	0.045
	T2D with renal complications	cML-MA	30	0.865 (0.549-1.362)	0.531
		MR-RAPS	30	0.863 (0.547-1.363)	0.529
		MR Egger	30	1.270 (0.524-3.078)	0.600
		Simple mode	30	1.122 (0.385-3.270)	0.835
		Weighted median	30	0.976 (0.523-1.821)	0.938
		Weighted mode	30	0.969 (0.460-2.039)	0.934
		MR-PRESSO	30	0.864 (0.572-1.307)	0.496
		Radial MR	30	0.863 (0.600-1.242)	0.435
	UACR in diabetics	cML-MA	20	1.237 (0.840-1.821)	0.281
		MR-RAPS	20	1.239 (0.841-1.825)	0.277
		MR Egger	20	1.119 (0.417-3.004)	0.826
		Simple mode	20	1.058 (0.323-3.465)	0.927
		Weighted median	20	1.147 (0.624-2.112)	0.658
		Weighted mode	20	0.686 (0.230-2.053)	0.509
		MR-PRESSO	20	1.236 (0.789-1.934)	0.366
		Radial MR	20	1.238 (0.760-2.017)	0.401
Serum iron	Diabetic nephropathy	cML-MA	13	0.898 (0.716-1.126)	0.352

	Outcome	Method	nSNPs	*OR* (95%*CI*)	*P*
		MR-RAPS	13	0.897 (0.716-1.124)	0.345
		MR Egger	13	1.065 (0.746-1.520)	0.736
		Simple mode	13	0.742 (0.345-1.596)	0.461
		Weighted median	13	0.970 (0.728-1.293)	0.837
		Weighted mode	13	0.982 (0.745-1.293)	0.897
		MR-PRESSO	13	0.897 (0.702-1.147)	0.405
		Radial MR	13	0.897 (0.659-1.221)	0.503
	eGFRcrea in diabetics	cML-MA	10	0.993 (0.963-1.024)	0.640
		MR-RAPS	10	0.993 (0.963-1.024)	0.647
		MR Egger	10	0.951 (0.909-0.995)	0.062
		Simple mode	10	1.003 (0.943-1.067)	0.917
		Weighted median	10	0.978 (0.943-1.014)	0.235
		Weighted mode	10	0.979 (0.949-1.010)	0.217
		MR-PRESSO	10	0.993 (0.961-1.026)	0.675
		Radial MR	10	0.993 (0.953-1.034)	0.737
	T1D with renal complications	cML-MA	13	0.988 (0.651-1.500)	0.956
		MR-RAPS	13	0.980 (0.649-1.481)	0.924
		MR Egger	13	1.343 (0.714-2.524)	0.380
		Simple mode	13	0.995 (0.255-3.885)	0.994
		Weighted median	13	1.067 (0.626-1.819)	0.811
		Weighted mode	13	1.160 (0.685-1.963)	0.591
		MR-PRESSO	13	0.980 (0.633-1.519)	0.930
		Radial MR	13	0.980 (0.533-1.803)	0.950
	T2D with renal complications	cML-MA	13	0.724 (0.503-1.042)	0.082
		MR-RAPS	13	0.719 (0.502-1.031)	0.073
		MR Egger	13	1.050 (0.616-1.790)	0.862
		Simple mode	13	0.672 (0.233-1.937)	0.476
		Weighted median	13	0.819 (0.515-1.303)	0.399
		Weighted mode	13	0.850 (0.541-1.334)	0.493
		MR-PRESSO	13	0.720 (0.486-1.067)	0.128
		Radial MR	13	0.719 (0.411-1.259)	0.271
	UACR in diabetics	cML-MA	11	1.009 (0.782-1.302)	0.947
		MR-RAPS	11	1.013 (0.787-1.303)	0.920
		MR Egger	11	1.096 (0.753-1.595)	0.644
		Simple mode	11	0.995 (0.534-1.854)	0.988
		Weighted median	11	0.871 (0.635-1.194)	0.390
		Weighted mode	11	0.890 (0.630-1.256)	0.521
		MR-PRESSO	11	1.013 (0.826-1.242)	0.904
		Radial MR	11	1.013 (0.726-1.413)	0.941
TIBC	Diabetic nephropathy	cML-MA	13	0.863 (0.754-0.989)	0.035
		MR-RAPS	13	0.864 (0.753-0.990)	0.035
		MR Egger	13	0.858 (0.724-1.016)	0.103
		Simple mode	13	1.041 (0.655-1.656)	0.866

	Outcome	Method	nSNPs	*OR* (95%*CI*)	*P*
		Weighted median	13	0.859 (0.739-1.000)	0.049
		Weighted mode	13	0.864 (0.740-1.009)	0.090
		MR-PRESSO	13	0.864 (0.771-0.968)	0.027
		Radial MR	13	0.864 (0.736-1.013)	0.096
	eGFRcrea in diabetics	cML-MA	9	1.008 (0.990-1.027)	0.359
		MR-RAPS	9	1.008 (0.990-1.027)	0.359
		MR Egger	9	1.001 (0.979-1.024)	0.917
		Simple mode	9	1.002 (0.941-1.067)	0.949
		Weighted median	9	1.006 (0.988-1.025)	0.514
		Weighted mode	9	1.006 (0.987-1.025)	0.557
		MR-PRESSO	9	1.008 (0.993-1.024)	0.306
		Radial MR	9	1.008 (0.973-1.046)	0.660
	T1D with renal complications	cML-MA	13	0.743 (0.579-0.954)	0.020
		MR-RAPS	13	0.743 (0.578-0.954)	0.020
		MR Egger	13	0.836 (0.613-1.140)	0.282
		Simple mode	13	0.851 (0.373-1.940)	0.708
		Weighted median	13	0.771 (0.583-1.020)	0.068
		Weighted mode	13	0.781 (0.590-1.033)	0.109
		MR-PRESSO	13	0.743 (0.603-0.916)	0.017
		Radial MR	13	0.743 (0.472-1.168)	0.222
	T2D with renal complications	cML-MA	13	0.925 (0.745-1.150)	0.483
		MR-RAPS	13	0.926 (0.746-1.151)	0.489
		MR Egger	13	0.847 (0.647-1.109)	0.253
		Simple mode	13	1.353 (0.620-2.950)	0.462
		Weighted median	13	0.892 (0.701-1.135)	0.351
		Weighted mode	13	0.903 (0.707-1.153)	0.429
		MR-PRESSO	13	0.926 (0.749-1.146)	0.495
		Radial MR	13	0.926 (0.605-1.419)	0.731
	UACR in diabetics	cML-MA	10	0.917 (0.790-1.066)	0.260
		MR-RAPS	10	0.918 (0.790-1.066)	0.262
		MR Egger	10	0.836 (0.683-1.024)	0.121
		Simple mode	10	0.794 (0.427-1.475)	0.484
		Weighted median	10	0.923 (0.779-1.093)	0.351
		Weighted mode	10	0.893 (0.764-1.043)	0.187
		MR-PRESSO	10	0.918 (0.772-1.091)	0.355
		Radial MR	10	0.918 (0.594-1.419)	0.708
TSAT	Diabetic nephropathy	cML-MA	9	1.098 (0.895-1.347)	0.369
		MR-RAPS	9	1.093 (0.894-1.337)	0.384
		MR Egger	9	1.145 (0.691-1.898)	0.615
		Simple mode	9	1.092 (0.651-1.833)	0.746
		Weighted median	9	1.107 (0.843-1.455)	0.465
		Weighted mode	9	1.134 (0.880-1.460)	0.359
		MR-PRESSO	9	1.093 (0.828-1.443)	0.548

Outcome	Method	nSNPs	*OR* (95%*CI*)	*P*
	Radial MR	9	1.093 (0.813-1.469)	0.571
eGFRcrea in diabetics	cML-MA	8	0.988 (0.962-1.015)	0.372
	MR-RAPS	8	0.988 (0.961-1.015)	0.374
	MR Egger	8	0.954 (0.911-0.998)	0.087
	Simple mode	8	0.996 (0.954-1.040)	0.854
	Weighted median	8	0.978 (0.948-1.010)	0.178
	Weighted mode	8	0.981 (0.950-1.013)	0.283
	MR-PRESSO	8	0.988 (0.967-1.009)	0.305
	Radial MR	8	0.988 (0.961-1.016)	0.417
T1D with renal complications	cML-MA	9	1.229 (0.841-1.796)	0.287
	MR-RAPS	9	1.208 (0.836-1.746)	0.315
	MR Egger	9	1.310 (0.553-3.101)	0.559
	Simple mode	9	1.186 (0.557-2.523)	0.670
	Weighted median	9	1.285 (0.792-2.087)	0.310
	Weighted mode	9	1.333 (0.845-2.102)	0.252
	MR-PRESSO	9	1.207 (0.751-1.939)	0.459
	Radial MR	9	1.208 (0.776-1.879)	0.427
T2D with renal complications	cML-MA	9	0.944 (0.677-1.316)	0.734
	MR-RAPS	9	0.928 (0.673-1.279)	0.646
	MR Egger	9	1.261 (0.564-2.819)	0.589
	Simple mode	9	1.267 (0.618-2.596)	0.536
	Weighted median	9	1.096 (0.709-1.693)	0.681
	Weighted mode	9	1.070 (0.696-1.645)	0.765
	MR-PRESSO	9	0.928 (0.582-1.480)	0.762
	Radial MR	9	0.928 (0.541-1.590)	0.792
	Radial MR (Outlier corrected)	8	1.045 (0.751-1.455)	0.792
UACR in diabetics	cML-MA	9	0.933 (0.751-1.159)	0.531
	MR-RAPS	9	0.933 (0.751-1.160)	0.534
	MR Egger	9	0.868 (0.600-1.255)	0.476
	Simple mode	9	0.898 (0.624-1.291)	0.576
	Weighted median	9	0.888 (0.683-1.154)	0.373
	Weighted mode	9	0.893 (0.693-1.152)	0.410
	MR-PRESSO	9	0.933 (0.840-1.038)	0.238
	Radial MR	9	0.933 (0.825-1.056)	0.305

TSAT, Transferrin saturation; TIBC, Total iron binding capacity; T1DM, Type 1 diabetes; T2DM, Type 2 diabetes mellitus; eGFRcrea, estimated Glomerular filtration rate by serum creatinine; UACR, Urinary albumin-to-creatinine ratio; SNP, single nucleotide polymorphism; *OR*, odds ratio; *CI*, confidence interval; IVW, inverse variance weighted; MR, mendelian randomization; cML-MA, constrained maximum likelihood and model averaging; MR-PRESSO, MR Pleiotropy RESidual Sum and Outlier tests; MR-RAPS, the Robust Adjusted Profile Score of MR.
