# Supplementary material for: Causal Associations of Iron Status With the Renal Function and Diabetic Nephropathy in Patients With Diabetes Mellitus: A Two-Sample Mendelian Randomization Study
Source: J Diabetes Res. 2025 Jul 30;2025:6658794. doi: 10.1155/jdr/6658794 (PMC12401605; doi:10.1155/jdr/6658794)
Supplement: Supporting Information 6 [file 6658794.f6.pdf]

MR-Egger method possesses the capacity to withstand potential pleiotropy and provide conservative estimations of results [1]. Weighted median method is a reliable approach for estimating causal effects and offers the significant advantage of accommodating up to 50% invalid instrumental variables [2]. CML-MA method, a MR technique that integrates machine learning and model averaging, is employed to address both correlated and uncorrelated pleiotropic effects. Importantly, it circumvents the need for assuming Instrument Strength Independent of Direct Effect (InSIDE), distinguishing itself from other Mendelian randomization approaches. Furthermore, cML-MA exhibits superior control over type I errors [3]. MR-RAPS method directly incorporates pleiotropic effects of genetic variants by utilizing a random-effects distribution, which is expected to yield favorable performance when the pleiotropic effects follow a normal distribution centered around zero [4]. Simple mode identifies the largest cluster to estimate the causal effect of SNPs, while the weighted mode assigns specific weights to each SNP [5]. MR-PRESSO outlier test was utilized with 10,000 distributions to detect and eliminate outliers, effectively addressing the issue of horizontal pleiotropy [6]. Weighted mode method offers a comprehensive assessment of the impact of different genotypes on outcomes by calculating the weighted average of each genotype. It effectively controls for variations in genotype frequency and ensures robustness and accuracy in the analysis [7].

1. Burgess S, Thompson SG. Interpreting findings from Mendelian randomization using the MR-Egger method. European journal of epidemiology. 2017; 32: 377-89.

2. Bowden J, Davey Smith G, Haycock PC, Burgess S. Consistent Estimation in Mendelian Randomization with Some Invalid Instruments Using a Weighted Median Estimator. Genetic epidemiology. 2016; 40: 304-14.

3. Xue H, Shen X, Pan W. Constrained maximum likelihood-based Mendelian randomization robust to both correlated and uncorrelated pleiotropic effects. American journal of human genetics. 2021; 108: 1251-69.

4. Liu S, Li F, Cai Y, Ren L, Sun L, Gang X, et al. Unraveling the mystery: a Mendelian randomized exploration of gut micr obiota and different types of obesity. Front Cell Infect Microbiol. 14: 1352109.

5. Qi W, Wang D, Hong Y, Yao J, Wang H, Zhu L, et al. Investigating the causal relationship between thyroid dysfunction diseases and osteoporosis: a two-sample Mendelian randomization analysis. Scientific reports. 2024; 14: 12784.

6. Verbanck M, Chen CY, Neale B, Do R. Detection of widespread horizontal pleiotropy in causal relationships inferred from Mendelian randomization between complex traits and diseases. Nature genetics. 2018; 50: 693-8.

7. Qian J, Zheng W, Fang J, Cheng S, Zhang Y, Zhuang X, et al. Causal relationships of gut microbiota, plasma metabolites, and metabolite ratios with diffuse large B-cell lymphoma: a Mendelian randomization study. Frontiers in microbiology. 2024; 15: 1356437.
